# Impacts of microplastics on immunity

**DOI:** 10.3389/ftox.2022.956885

**Published:** 2022-09-27

**Authors:** Wenjie Yang, Nahar Jannatun, Yanqiao Zeng, Tinghao Liu, Guofang Zhang, Chunying Chen, Yang Li

**Affiliations:** ^1^ Laboratory of Immunology and Nanomedicine, Shenzhen Institute of Advanced Technology, Chinese Academy of Sciences, Shenzhen, China; ^2^ CAS Key Laboratory for Biomedical Effects of Nanomaterials and Nano Safety, National Centre for Nanoscience and Technology of China, Chinese Academy of Sciences, Beijing, China; ^3^ GBA Research Innovation Institute for Nanotechnology, Guangzhou, Guangdong, China

**Keywords:** microplastics, immune response, phagocytosis, immunotoxicity, protein-corona, combined exposure

## Abstract

Most disposable plastic products are degraded slowly in the natural environment and continually turned to microplastics (MPs) and nanoplastics (NPs), posing additional environmental hazards. The toxicological assessment of MPs for marine organisms and mammals has been reported. Thus, there is an urgent need to be aware of the harm of MPs to the human immune system and more studies about immunological assessments. This review focuses on how MPs are produced and how they may interact with the environment and our body, particularly their immune responses and immunotoxicity. MPs can be taken up by cells, thus disrupting the intracellular signaling pathways, altering the immune homeostasis and finally causing damage to tissues and organs. The generation of reactive oxygen species is the mainly toxicological mechanisms after MP exposure, which may further induce the production of danger-associated molecular patterns (DAMPs) and associate with the processes of toll-like receptors (TLRs) disruption, cytokine production, and inflammatory responses in immune cells. MPs effectively interact with cell membranes or intracellular proteins to form a protein-corona, and combine with external pollutants, chemicals, and pathogens to induce greater toxicity and strong adverse effects. A comprehensive research on the immunotoxicity effects and mechanisms of MPs, including various chemical compositions, shapes, sizes, combined exposure and concentrations, is worth to be studied. Therefore, it is urgently needed to further elucidate the immunological hazards and risks of humans that exposed to MPs.

## Introduction

Plastics include a wide range of synthetic/semi-synthetic materials that use polymers as their main component. The first-ever artificial plastic “Parkesine” was introduced to the world in 1862 by Alexander Parkes, but it did not achieve commercial success. Further, John Wesley Hyatt improved the Parkesine and named it “celluloid,” which could imitate substances like tortoiseshells, horn, linen, and ivory and is used for producing various products such as table tennis balls and office equipment (Nicholson and Leighton, 1942). In the past decades, the applications of plastics have increased exponentially because the improved plastics are inexpensive, light-weight, durable, and flexible. However, in recent years, the pollution caused by the misuse of plastic has become more serious. The annual global production of plastic is estimated to be 270 million tons; meanwhile, the corresponding waste production is around 275 million tons. Such heavy plastic pollution risks that about eight million tons of plastic may enter the global water bodies every year, and estimated that there might be 10,000–100,000 tons of plastic in the surface water and even in the greater depths of the seafloor (Jambeck et al., 2015). Microplastics (MPs) are plastic fragments of less than 5 milimeters(mm), while nanoplastics (NPs) are smaller plastic fragments (≤100 nm) (Cole et al., 2011; Amobonye et al., 2021; Gigault et al., 2021). In this paper, MPs referred to any plastic particles below 5 mm in size. MPs are usually derived from plastics in all aspects of daily life and may eventually enter the environment as waste, such as the ocean, tap water, and soil (Collignon et al., 2014). MPs in marine are usually divided into two main types: primary MPs (*e.g.*, fabric microfibers, microbeads, and plastic pellets) and secondary MPs (produced by natural degradation of larger plastics) (Cole et al., 2013; Botterell et al., 2019). It has been detected more than 10,000 MP/m^3^ in surface water, 52–13,832 beads/m^2^ in marine sediments (Norén, 2007; Van Cauwenberghe et al., 2015), and up to 2,000 microparticles/m^2^ in the deep oceanic sediments at a depth of 5,000 m (Fischer et al., 2015). Plastic pollution caused by MPs on land freshwater also under concern. Tap water from 159 global sources was tested, and 81% were found to contain MPs (Kosuth et al., 2018). Tests on 259 individual bottles of water from 11 different brands and 27 different batches, and the results indicated that 93% of the tested samples contained MPs (Mason et al., 2018). Researchers also detected MP fragments in honey, beer, salt, sugar, fish, shrimp, and bivalve organisms (Liebezeit and Liebezeit, 2014; Li et al., 2015; Neves et al., 2015). In addition, MPs are found up to 593 particles/kg of soil sample (Scheurer and Bigalke, 2018). The hazard of MPs arises from their slow degradation in the environment, which may continue over hundreds to thousands of years. Thus, threatening assessments to organisms and humans are also highly needed.

**FIGURE 1:**
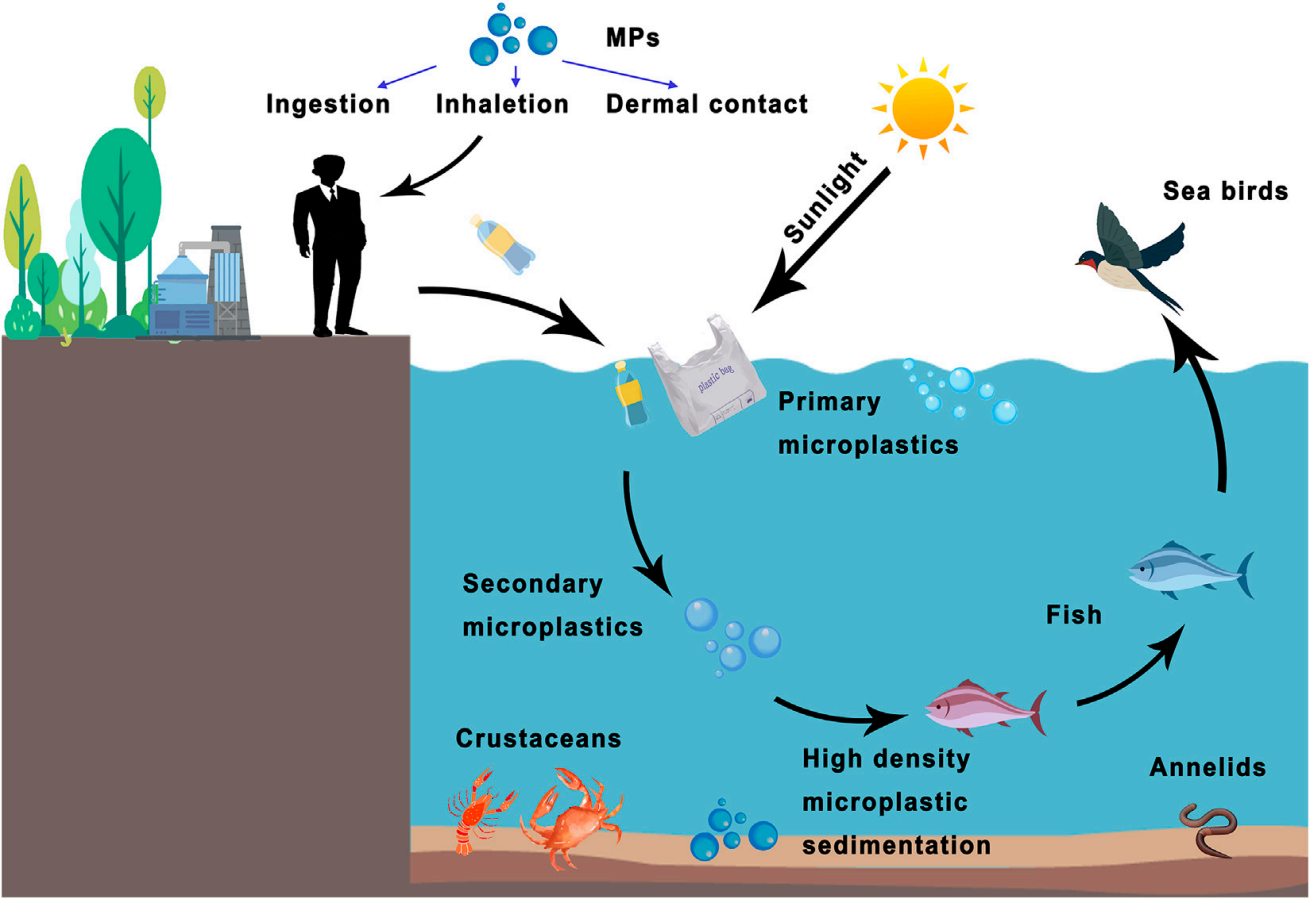
The bioconcentration of MPs in living organisms and potential human exposure ways. Plastics produced by humans are degraded into primary and secondary microplastics through seawater or sunlight. MPs can be swallowed by fish and passed along the food chain. Other high-density MPs become sediments that endanger marine ecology. Humans can be exposed to MPs through various routes (*e.g.*, ingestion, inhalation, or dermal contact).

MPs are exposed to human beings by multiple routes, *e.g.*, ingestion, inhalation, and dermal contact, that inevitably translocate into organisms and humans tissues (Sykes et al., 2014; Wright and Kelly, 2017; Mason et al., 2018). It is estimated that, the average human ingestion of MPs is about 0.1–5 g per week (Senathirajah et al., 2021). Humans using bottled water are assumed to ingest extra 90,000 particles compared to those who solely drink tap water (ingesting only 4,000 extra particles) (Cox et al., 2019). It can be extrapolated that ordinary adults are exposed up to 94,000–114,000 MPs per annum, among them up to 48,000–69,000 MPs through inhalation (Cox et al., 2019). In an outdoor atmosphere, especially in densely populated, high-traffic places (*e.g.,* dormitories and offices) (Llorca and Farre, 2021), or even indoors (*e.g.*, the use of air conditioning) (Vianello et al., 2019; Zhang et al., 2020; Soltani et al., 2021), humans are more likely to inhale MPs. It should be further noticed that the fuel-based MPs can be oxidized and decomposed under light, heat, and microorganisms, which causes the release of chemicals into the environment, *e.g.,* tetrabromobisphenol A (TBBPA), polybrominated diphenyl ethers (PBDE), polycyclic aromatic hydrocarbons (PAHs) (Hirai et al., 2011; Li et al., 2021b). Polystyrene (PS) is widely used daily for food packaging, surface coatings, drinking bottles, and other household products involved in bisphenol A (BPA) and phosgene. BPA is a potent carcinogen and can be released into the water and environment through plastic degradation, inducing the disruption of our endocrine functions upon intake, which implies that humans may readily be exposed to these chemicals in various ways due to the ubiquity of MP wastes (Van Cauwenberghe et al., 2015). Thus, the influence of MPs on aquatic (Botterell et al., 2019) and terrestrial organisms (Bradney et al., 2019) should be concerned. MPs have been reported to affect the behavior, physiology, and metabolism of fishes and mollusks (Van Cauwenberghe and Janssen, 2014; Mattsson et al., 2015; Smith et al., 2018)*.* Zebrafish is used as an aquatic model to assess the ecotoxicology and the impact of the biological processes (Harris et al., 2014; Bhagat et al., 2020). MPs larger than 200 μm were observed in the digestive tracts (no MPs were found in muscle) from 166 of 240 human edible marine organisms’ samples, which was the most abundant in the carnivore digestive tract (Alfaro-Nunez et al., 2021). Meanwhile, nine types of MPs have been detected in human feces, indicating that the food chain is a significant source of MPs entering the human gastrointestinal tract (Figure 1) (Schwabl et al., 2019; Issac and Kandasubramanian, 2021). Thus, accurate monitoring of MPs in the environment is critically important for understanding the impact of MPs on humans and other organisms (Shoopman and Pan, 2021). The European Commission has allocated a budget of 30 million Euros to form a multidisciplinary team named CUSP to carry out a 5-years project to understand the environmental and biological consequences of MP exposure, as well as the potential risks to humans (Inoue et al., 2001; Flint et al., 2012).

Under this unoptimistic situation, where the available water resources to human beings, marine living organisms and aquatic products contain plastic contamination, it is important to have immune defense system against foreign plastics. Neutrophil activation, epithelial detachment, mucous hyper secretion, and immune responses were noted after MPs exposure in zebrafish (Limonta et al., 2019). Immune activation and gut microbiota dysbiosis following ingestion have also been revealed in fish (Li et al., 2021a) and mouse (Sun et al., 2021), indicating potential immune activation and toxicity in vertebrate. Human exposure to MPs can also result in the activation of immune defensive responses. Long-term exposed workers to polypropylene microfiber were more likely to be at risk for interstitial pneumonia and respiratory inflammation (Atis et al., 2005). Importantly, MPs are present in human blood as high as 1.6 μg/ml, which cannot be ignored for its potential hazardous effects (Leslie et al., 2022). Due to the natural complex composition and surface properties of MPs, other substances or pathogens of the environment are readily adsorbed/attached to the surface, leading to unexpected consequences. In such cases, MPs become the carriers of pollutants or pathogens for their quickly invading against our bodies. Recently finding reported the content of SARS-CoV-2 has a significant positive correlation with MPs in the aerosol collected near a medical center in Latin America with unknown consequences (Amato-Lourenco et al., 2022b). They imply that air source MPs may become the carriers of SARS-CoV-2 for their spreading and transmission in the current COVID-19 pandemic.

The immune system is made by various cells and factors protecting us from invading foreign substances, including pathogens and MPs. Upon invading, innate immune cells such as macrophages and neutrophils will be recruited, and the inflammatory cytokines (*e.g.*, IL-1β and TNF-α) will be released to solve the potential danger (Lu et al., 2021). However, there is still not enough information and assessment regarding the interaction of MPs with immune systems and the potential immuno-safety issues. In addition, although MPs are widely present in the environment and readily interact with the potential pathogens that may be harmful to humans, the potential combo-exposure effects on human health have not been noticed, which may cause serious consequence. Hence, in-depth studies of MPs exposure, bio-distribution, and their interaction with human immunity are in urgent need. Thus, the following review will carefully assess the short and long-term immunosatety after MPs exposure and the MP-pathogen co-exposure potential.

## The effect of biocorona and the combined exposure of ubiquitous microplastics

The major challenge in understanding the toxicity of MPs is exploring the relationships between MPs characteristics and the bio-microenvironment. Like nanomaterials, the biocorona will also be formed when MPs are presented in a rich plasma environment. Several immune proteins in the cellular environment can recognize and attach to MPs, and the size and surface charge of particles influence the corona. The MP-corona may be comprised of immunoglobulins (IgGs), apolipoproteins, complement proteins, acute-phase proteins, and coagulation proteins (Lundqvist et al., 2008). MPs attach to cell surface receptors via the corona; hence corona proteins control the fate of MPs inside the cells. Corona formation is thought to be one of the mechanisms that lessen the toxic impact of MPs. Eco-corona formation was described earlier in algal exudates of Chlorella sp (Tunali et al., 2020). In this study, three differently functionalized polystyrene MPs (PS MPs) (*e.g.*, aminated (NH_2_-PS MPs), carboxylate (COOH-PS MPs), and unmodified MPs) were used to facilitate eco-corona formation and to significantly decline the oxidative stress and toxic effects, which the response process depended on the MP’s surface (Natarajan et al., 2020). However, the corona facilitates MP translocation to almost every organ, which may lead to their uncontrolled impact and potential health hazards (Mowat, 2003; Mohr et al., 2014; Wright and Kelly, 2017; Abihssira-Garcia et al., 2020). It may be why MP accumulation in the gut and their increased cytotoxicity (Krug and Wick, 2011; Walczak et al., 2015). Through the exploration of the gold nanoparticles, it was found that the internalization by murine macrophages was directly correlated with the number of proteins adsorbed onto the particle surface (Walkey et al., 2012). These proteins act as chemical stimuli for the attachment and internalization of metal, polymer or MPs into macrophages, which is the process governed by Fc or scavenger receptor-mediated phagocytosis (Walkey et al., 2012).

Protein-corona formulation is a crucial factor inducing the cellular internalization of MPs entering cells via clathrin-coated vesicles, macro-pinocytosis, and phagocytosis. This transport causes MPs to be interacted with various biomolecules due to their small sizes and greater surface-to-mass ratio. In nanomaterials, black phosphorus bind to the intracellular PLK1 kinase for their inactivation, affecting the cell cycle process and in trun to trigger cellular apoptosis (Shao et al., 2021). Thus, MP may also induce cell stress by attaching to proteins or other bio-molecules. In addition, MPs acquire new biological characteristics after corona formation, which may allow them to escape immune surveillance, extend circulatory half-life and interfere with cellular and molecular mechanisms. Corona-coated MPs decreased red blood cell (RBC) agglutination compared with pure MP; thus, some proteins may act as opsonins and facilitate plastic-fragment internalization and MP-cell interactions (Markiewicz et al., 2018). They may undergo secondary/tertiary conformational changes after combination with proteins, which can convert these toxic complexes into immunogenic structures that may induce autoimmune reactions, as seen for MPs, which affect thyroid metabolism (Mariussen and Fonnum, 2003; Mowat, 2003; Goy-Lopez et al., 2012; Xu et al., 2019). Studies have found that virgin-coronated and isolated MPs (13–600 nm) exert severe toxicity on human blood cells and Allium cepa root tips, respectively (Deng et al., 2017b; Gopinath et al., 2019). These multi-layered coronated MP complexes induced protein-based coalescence in MPs, which caused conformational alterations and denaturation of corona protein, to make it a bio-incompatible molecule and resulting in genotoxicity and cytotoxicity in human blood cells. Meanwhile, virgin-MPs and isolated MPs from facial scrubs hindered the Allium cepa root growth and induced chromosomal aberrations during ring formation and C-mitotic and chromosomal breaks (Deng et al., 2017b; Gopinath et al., 2019).

However, the protein-corona coating of MPs may induce the Trojan horse effect, by which these particles can easily get internalized without interacting with membrane receptors. Thus, the idea that plastic exhibits an “inert” nature may cause a toxicological impact, having implications for the environment and human health (Ramsperger et al., 2020; Yong et al., 2020). Several unknown mechanisms in the human body need to be studied. For example, what types of corona formed on different kinds of MPs, whether toxic molecules can bind the MP, whether the corona from the cellular environment is in favor of MP-uptake through the intestines, and whether the corona’s composition changes in the human/mouse organs or blood and for how long it survives (Cordani and Somoza, 2019; Tinnevelt et al., 2019).

In addition to the adverse effects of plastics and corona, their toxicological effects are also closely related to the degradation of the plastic itself and other pollutants adsorbed on its surface. Studies have confirmed that MPs can also be used as carriers to form biological ligands and other metals such as zinc and copper or environmental pollutants, which helps them be transported into organisms (Liu et al., 2021). If some heavy metals such as arsenic, cadmium, and Mercury are adsorbed on the surface of MPs, the toxicological and mortality risk will be much higher. Some genes in the liver are up-regulated, and the damage is more harmful when MPs adsorb chemicals (Rainieri et al., 2018; McKechnie and Blish, 2020). The influence of MPs’ surface roughness (Holmes et al., 2012; Blanco-Melo et al., 2020), texture (Napper et al., 2015; Coates and Soderhall, 2020), and temperature (Crawford and Quinn, 2017) will affect the affinity of contaminant on their surface. Researchers used two synthetic MPs (glassy PS and rubbery PE) and a natural MP (silica glass) spiked with hydrophobic organic chemicals (HOCs) as vectors to explore the transfer, distribution, and biological effects of chemical pollutants in fish, and found that the HOCs relase from MPs, activated intestinal metabolism and accumulated in the liver (Asmonaite et al., 2020). Compared with silica particles, PS and PE MPs have higher chemical transfer and a more significant accumulation of HOCs in the muscle (Asmonaite et al., 2020). Also, with the continuous degradation of MPs, the gradual aging of the surface increases its surface area, which makes it more easily adsorb and release chemical pollutants. For example, phthalates and BPA are the key raw materials used for PVC synthesis. When BPA is released into the environment and ingested, it destroys amino acid metabolism, steroid metabolism, and energy metabolism pathways and induces oxidative stress (Gomez and Gallart-Ayala, 2018). It suggested that studying the potential effects of combined MP exposure on human health is necessary.

## The risk of microplastics-pathogens interaction on health

### The microbiota

MPs may enter the human body via three important routes: inhalation, ingestion, and skin exposure through urban dust and fumes from industrial, textile, or rubber tire pollution (Rahman et al., 2021). Skin exposure to MPs through wounds, sweat glands, or hair follicles, and the most common routes of human exposure to MPs are via the food and water chains (Carbery et al., 2018). A considerable number of MPs enter the human body through seafood and the environmental components (Camacho et al., 2019). Thus, the gastrointestinal tract (GI) directly exposed to MPs and their complexes. Mice expose to positively charged NH_2_-PS negatively charged COOH-PS, and pristine polystyrene PS MPs of two sizes (70 nm and 5 μm in diameter) for 28 days respectively, the intestinal barrier was damaged after exposure, and led to significant gut microbiota dysbiosis (Qiao et al., 2021), suggesting that gut microbiota plays a vital role in protecting gut health against MP invasion and that MPs may affect human gut health in the same way. The interactions of food-related MPs following ingestion have attracted considerable attention. Some non-absorbable particles directly colonize the surface of intestinal epithelial cells and interact with gut microbes (Radziwill-Bienkowska et al., 2018). Nanomaterials (ZnO, TiO_2_, etc.) have been shown to influence compositions and metabolic activities of gut microbiota after ingestion (Taylor et al., 2015; Xia et al., 2017). However, although TiO_2_ and SiO_2_ are less absorbed in the body, it is easy to accumulate in the intestinal lumen (Chen et al., 2017). The shape and size of MPs are key factors of influencing gut microbiota; for example, Ag-nanoparicles change the gut microbiota composition (Perez et al., 2021) and affect the human gut microbiota structure after ingestion to promote the growth of pathogenic bacteria (Das et al., 2014). Thus, assessing their effects in more appropriate doses and time for human health is still a necessary.

MPs have attracted more and more attention by affecting gut microbiota composition to promote the development of some diseases, such as CRC, IBD, and obesity (Belkaid and Hand, 2014; Burcelin, 2016; Lazar et al., 2018). Thus, MPs in human feces explored a clear and positive association with IBD patients (Yan et al., 2022) suggesting potential health risks and disease development after GI exposure to MPs. Whether MPs further participate in the disease development in gut microbiota dysbiosis or the disease development affects the MPs’ retention in the body remains an area worth studying. The abundance of *Staphylococcus* in gut microbiota is significantly increased and induces the gut inflammatory response after the mouse fed with a high concentration of polyethylene MPs (Li et al., 2020). Related research has been carried out on zebrafish, which are used as a model to reveal the impacts of MPs on aquatic biological processes, ecotoxicology, and disease development pathways (Harris et al., 2014; Bhagat et al., 2020). Different MPs’ shapes accumulate in the gut differently, within the most accumulation is fiber MPs’. At the same time, intestinal dysbiosis and changes in specific flora caused by MPs’ exposure exacerbate intestinal inflammation (Qiao et al., 2019). Therefore, understanding the interaction between MPs on gut microbiota and disease development is crucial to exploring the risk assessment of MPs on human health.

### Pathogens

The surfaces of MPs from the natural environment affected by various factors, which are natural attachment carriers for other chemical pollutants, pathogens, and substances. After aging treatment of PS and PET MPs under experimental conditions, the adsorption capacity of copper [Cu (II)] from environmental pollutants is robust, especially for MPs’ exposed to high temperatures for a long time (Wang et al., 2022). This adsorption capacity also effectively reduces the volatilization of anthracene and pyrene, which affects the photoinduced toxicities of anthracene and pyrene to Phaeodactylumtricornutum and Selenastrumcapricornutum (Huang et al., 2022). Moreover, heavy metal substances from the environment (*e.g.,* copper, arsenic, lead, zinc, cadmium, and chromium) can be adsorbed on the surfaces of MPs (Gao et al., 2021). When these MPs attached to these chemicals are ingested, they will inevitably interact with the immune system, posing significant risks to health.

In addition, organisms (*e.g.*, bacteria and viruses) can also be affected by MPs to pose a threat to human health. *Helicobacter pylori* attach to the surfaces of MP fragments to form “biofilms” (Tong et al., 2022). Mixing MPs with *Helicobacter pylori* (*H. pylori*) significantly accelerates the infection of *H. pylori* and promotes the inflammation response of stomach and intestinal tissues, resulting in more severe inflammatory cell infiltration (Tong et al., 2022). This study provides strong evidence that bacteria can attach to MPs’ surfaces and exacerbates the development of inflammation. Furthermore, during the novel coronavirus pandemic caused by SARS-CoV-2 that broke out in 2019, it was found that SARS-CoV-2 can exist stably on plastic surfaces at room temperature. By air sampling, it is shown that SARS-CoV-2 can be combined with aerosols (including MPs in the air), and the virus remains infectious for a few hours, which help the virus be transported in the air over long distances (Zhu et al., 2020). Recent studies have shown that MPs produced by plastic degradations are more conducive to the binding of SARS-CoV-2, making it easier for people to transmit the virus by inhalation (Amato-Lourenco et al., 2022a). SARS-CoV-2 can attach to the surface of MPs and utilize MPs as a carrier for intracellular transport, in consequence leading an increase infection of the virus (Zhang et al., 2022a). The interaction of MPs and the pathogens may threaten human health and cannot be ignored. Meanwhile, MPs may also prevent pathogens invasion if the MP occupied the virus-host binding site as nanomaterials (Zhang et al., 2022b). Thus, Further study of the combination of these pathogens with MPs and the potential impact on humans should be further studied.

## Internalization of microplastics

**FIGURE 2:**
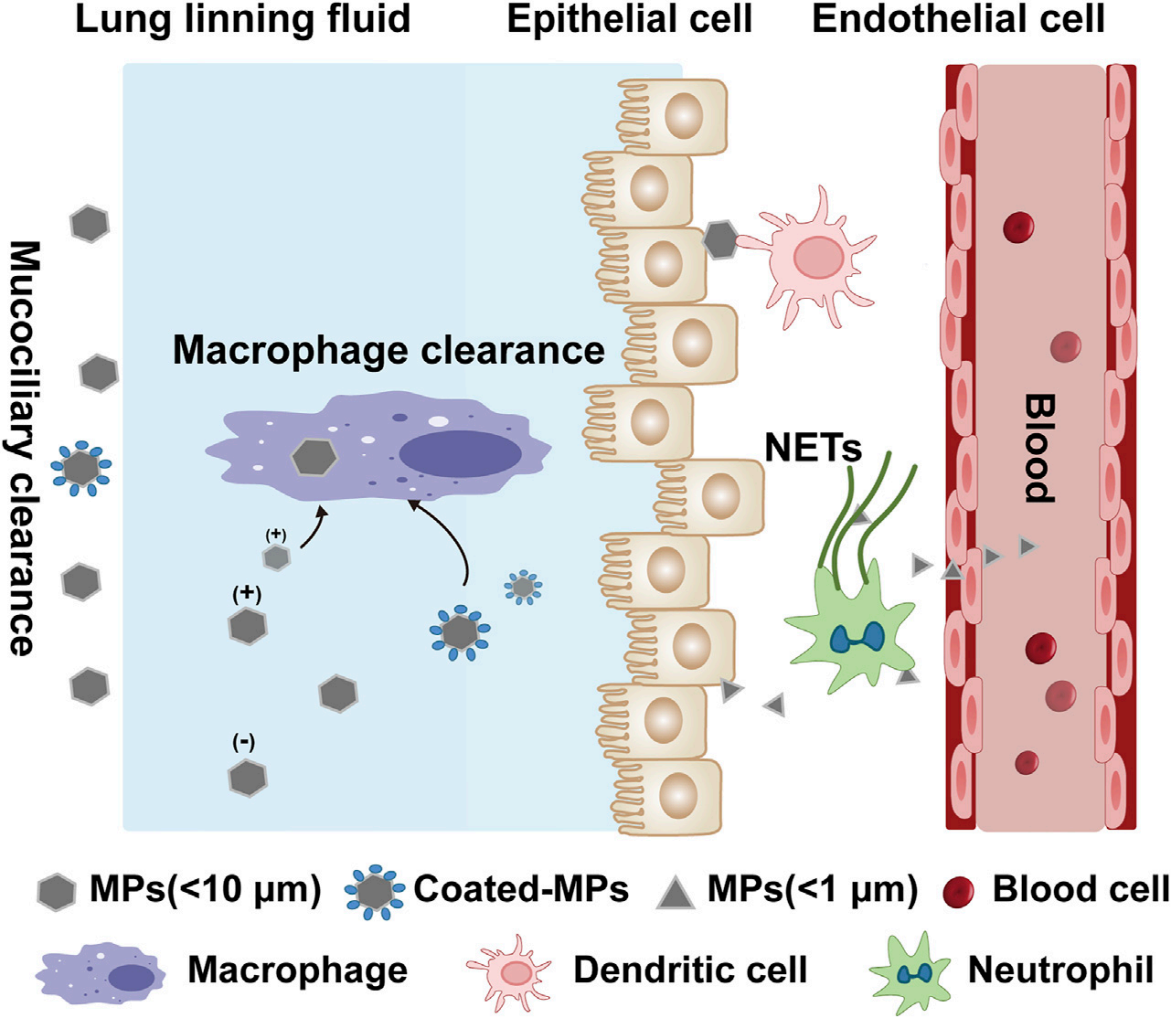
Potential internalization and clearance mechanisms of microplastics in the lungs. The lung lining fluid (surfactant and mucus) reduces the chance of microplastic displacement. Particles <10 μm are cleared by mucociliary, while particles <1 μm are uptaken through the epithelium and also may penetrate the thin lung lining fluid and contact the epithelium, and circulate and metastasize through diffusion or active cellular uptake. The surface charge and surface-molecular interactions of different MPs affect the immune cell clearance. For macrophage, positive charged MPs and coated-MPs (combined with protein or other substances) are more likely to interact with the cell membrane. Other immune cells are also involved in the defenses against MPs, such as antigen presentation by dendritic cells and the trapping and phagocytosis of MPs through the release of NETs by neutrophils, causing immune activation.

There is a continual battle between immune cells and external pathogens. The lungs and intestines are the most important organs that directly communicate with the external environment. In the lungs, large MPs are cleared by mucociliary clearance, while very small MPs (especially <1 μm) may penetrate through bronchial epithelial cells and contact endothelial cells, enter the circulatory system by passing through endothelial cells (Wright and Kelly, 2017; Zhang et al., 2021) (Figure 2). Inhaled MPs exhibit toxicity in human alveolar epithelial A549 cells (Forte et al., 2016). After being endocytosed by A549 cells, MPs inhibit the cell activity by blocking the cell cycle, promoting apoptosis, and up-regulating the expression of inflammation-related genes (Xu et al., 2019). Unlike the lungs, M cells in the Peyer’s patches of the intestine uptake MPs through endocytosis and transport them to the mucosal lymphatic tissues (Mowat, 2003). These plastic particles can disturb the signal pathways and gene expression (Deng et al., 2017b). Endocytosed MPs can also interfere with the extracellular transport pathway of intracellular transporters, the expression of intracellular receptors, and the transduction of signal pathways (Yong et al., 2020). Endocytosed MPs also can stimulate the autophagy process (Cordani and Somoza, 2019; Feng et al., 2020). Inhaled and ingested MPs first enter the GI or lungs, then break through the air-blood barrier or damage the gastrointestinal mucosa. Moreover MPs can enter major organs such as the liver, kidney, brain and the blood circulation (Yang et al., 2019; Shen et al., 2022), activate the innate immune response, and lead to immune system dysfunction in severe cases.

Numerous cells interact with MPs that penetrate into the epidermal layer. The number of absorbed particles in this process depends on key factors such as the particle size, surface charge, and functionality of the particle and the entity (proteins, carbohydrates, and phospholipids) they encounter (Yee et al., 2021). Some MPs are small enough to easily pass through biological membranes, making them even more hazardous. During the transport of MPs through the cell membrane, the particles may be endocytosed (inactive permeation) or interact with membrane channels or transport proteins (Mani and Pandey, 2019). Several endocytic mechanisms are observed, such as phagocytosis, macro-pinocytosis, and clathrin-/caveolae-mediated endocytosis (Mani and Pandey, 2019). This process leads phagocytes to uptake negatively charged MPs, which may behave as bacterial mimetics (Frohlich, 2012). The internalization of carboxylated polystyrene (PS) MPs (diameters: 40 and 200 nm) are observed in HeLa (cervical cancer), A549 (lung carcinoma) and 1321N1 (brain astrocytoma) human cell lines (dos Santos et al., 2011). The uptake processes rely on the active energy-dependent. Interestingly, the way different cells take up is not the same. The uptake of MPs in HeLa and 1321N1 cells was affected by actin depolymerization, while A549 cells more strongly inhabited MPs uptake after microtubule disruption by genistein (dos Santos et al., 2011). The phagocytosis of immune cells from the intestinal, blood, brain, and kidneys is dissimilar for different MPs. Immune cells from three different tissues were exposed to high, medium, and low concentrations of light-weight polyethylene microplastics (PE MPs) and yellow polystyrene plastic microplastics (PS MPs). The results showed that PE MPs were more accessible to uptake and accumulate than PS MPs, proving that phagocytic ability is related to the type of MPs (Abihssira-Garcia et al., 2020). Polystyrene MPs (200 nm) internalized and accumulated by mouse macrophages (RAW264.7) in the cytoplasm in a time-dependent manner; which significantly altered the ability of lysosomes to maintain acidic pH, causing lysosomal damage in macrophages (Florance et al., 2021). In the interaction of immune cells with MP uptake, positively charged MPs are more easy to permeate the cell membrane relative to negative and neutral MPs (Panariti et al., 2012). In addition, neutrophil extracellular traps (NETs) has the ability to capture nanoparticles forming macroaggregates, and then to facilitate the phagocytosis by neutrophils or macrophages (Bilyy et al., 2020). MPs may also have the similar phenomenon but not yet been descr. Protein corona formulation and combined exposure to the environment are also critical factors for the internalization of MPs for immune activation (Figure 2).

## Immune response after microplastic uptake

The innate immune system is the first line of defense against dangerous and harmful microbes. Innate immunity is also called the “non-specific” immune system, as it responds to all foreign organisms similarly. Phagocytes, especially white blood cells (leukocytes), are scavenging machines of innate immunity. These cells engulf and “digest” the antigens, whose information is transferred to the surface of the scavenger cells and then recognized by the adaptive immune system (McKechnie and Blish, 2020). The interleukin one family cytokines play an important role during this process (Zhang et al., 2022c). Neutrophils are the most rapidly responding immune cells in the immune system and can form phagosomes that non-specifically kill foreign pathogens. Therefore, they play an essential role in the immune system to prevent the immunotoxicity of the body. MPs derived from marine fiber fragments are often ingested by fish and accumulated after ingestion, *e.g.,* polyethylene, polypropylene, and polystyrene MPs induce the release of NETs and increase the degranulation of primary granules of neutrophils (Greven et al., 2016; Tanaka and Takada, 2016). That means MPs affect the fish’s behavior, physiology, and metabolism and interfere with the disease resistance in fish populations upon MP accumulation (Mattsson et al., 2015). *In vitro* neutrophil function assays showed that engulfing 7.3 μm MP resulted in massive (50–97% of the total neutrophils) cell death within 45 min, and phagocytosis of MPs (49–880 nm) led to 49–53% neutrophils fast death, and the MPs were re-released from dead cells (Tinnevelt et al., 2019; van Staveren, 2019). Interestingly, the phagocytosis of 880 nm MPs by the surviving neutrophils did not impair the bacterial phagocytosis function of these immune entities, and co-phagocytosis of bacteria and MPs did not hinder the immunological function of neutrophils. (Tinnevelt et al., 2019; van Staveren, 2019). These results provide a striking basis for future research on the immunotoxicity of MPs of humans.

After the uptake of MPs, immune cells make rapid and effective modulation in transcriptional levels, from enzymatic activities to cytokine release. For example, after exposure to PS MPs, the levels of IL-1α, IL-1β, and interferons in the zebrafish gut were significantly up-regulated (Jin et al., 2018). Following this, there were several alterations to zebrafish intestinal mucosa (*e.g.*, epithelial detachment, mucous hypersecretion, and higher neutrophil activation). Also, the immune response, epithelial integrity, and lipid metabolism-related genes are regulated (Limonta et al., 2019). Not only in fish, but the mice model exposed to different PE MPs, high concentrations of PE MPs significantly increased the composition and diversity of the intestinal microflora, causing inflammation of the small intestine via the expression of high levels of TLR4, AP-1, and IRF5 (Li et al., 2020). The level of IL-1α in the serum also increased significantly, and Th17 and Treg cells in CD4^+^ T cells are reduced after MP exposure (Li et al., 2020). The change in most immune-related factors after exposure to MPs includes hemocyanin (Hc), alkaline phosphatase (AKP), phenol oxidase (PO), lysozyme (LSZ), and acid phosphatase (ACP) in Chinese mitten crab (Eriocheir sinensis) (Liu et al., 2019). The Hc and LSZ gene expression showed a consistent trend with the corresponding changes in enzyme activities. It showed the effect of MPs on immune response by affecting immune enzyme activity through immune-related gene expression, proving that this immune response is non-specific (Liu et al., 2019). MPs exposure disrupt the immune response, including inhibiting Ca^2+^ signaling and apoptosis-related molecular pathways such as NF-κB (Tang et al., 2020). These studies have shown that the activation is closely associated with MP uptake, and this connection may affect adaptive immunity and innate immune responses. Researchers have found that 50 nm PS-MPs were easier to be internalized by murine splenocytes than 20 nm MP, resulting in decreasing of cell viability and increasing of cell apoptosis. This induced an obstruction on T cell differentiation by inhibiting PKCθ/NFκB and IL-2R/STAT5 signal pathway, which may increase the risk of infection and cancer (Li et al., 2022).

Researchers pay more attention to various biological and immunological effects after MP exposure. Amino-modified polystyrene MPs (NH_2_-PSMPs) have been used as a model to study the impact of MPs on marine bivalves due to the strong immune defense of shellfish against MPs (Auguste et al., 2020). After 24 h of short-term MPs exposure, the immune function of mussels (*M. galloprovincialis Lam.*) is significantly affected. The acidification of lysozyme and membrane destabilization decreased in circulating hemocytes, and serum lysozyme activity increased, indicating degranulation. After the second exposure, the absorption rate of NH_2_-PSMPs by cells is much lower, and the expression of stress and immune related genes are significantly reduced. However, the bactericidal activity of the whole hemolymph is increased considerably, indicating that shellfish’s immune compensation mechanism towards MP is established (Auguste et al., 2020). Polyvinylchloride (PVC) and polyethylene (PE) can also produce oxidative stress that induces immune protein dysfunction in head-kidney leucocytes (HKLs) (Espinosa et al., 2018). Providing oral administration of PE MPs to mice, the level of neutrophils in the blood increased, while the levels of white blood cells and lymphocytes decreased significantly. At the same time, PE MP particles migrated to the surface of mast cells in the stomach of mice, and the organelles, including mitochondria in the spleen cells, displayed abnormal accumulation (Park et al., 2020). The number of mature dendritic cells (CD11b−/CD11c+) decreased, while the ratio of CD4+/CD8+ significantly increased in a dose-dependent manner, which indicates that T helper cells play an essential role after PE MPs exposure. In addition, it is worth noting that the parental PE MPs treatment also affected the lymphocyte subsets in the spleen of the offspring (Park et al., 2020). Researchers used PVC and another widely used plastic acrylonitrile butadiene styrene (ABS) to explore the effect of MPs on human immune cells. When PBMC is exposed to MPs for 4–5 days, the cellular immune response is activated. ABS and PVC induced the production of IL-6 and TNF-α, respectively, while both inhibited the release of histamine (Han et al., 2020). However, TNF-α and IL-2 decreased upon increasing the PVC MPs concentration, indicating that exposure to MPs can cause an immune response in human cells (Han et al., 2020). Current research on MPs and the adaptive immune system is not in-depth, and the interaction mechanism of MPs at the cellular level is still unclear. Therefore, more studies are needed to explore the potentially harmful effects of MPs on human health.

**FIGURE 3:**
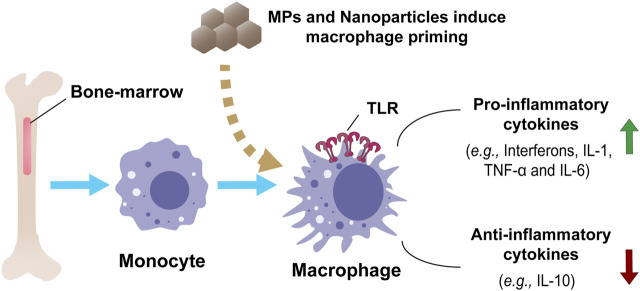
*In vitro* model of macrophage priming and innate immune functions in human monocytes and monocyte-derived macrophages by nanoparticles or MPs. Bone-marrow-derived macrophages show an innate response in producing and regulating inflammatory and anti-inflammatory cytokines.

The epigenetic programming of the innate immune cells (macrophages, monocytes, and natural killer cells) is reported to induce memory properties in this subset of cells, which is called “trained innate immunity” or “innate immunity memory” (Boraschi and Italiani, 2018). MPs and nanoparticles have the potential to induce the generation of innate immunity memory. Pristine graphene directly triggers the enhanced secondary response to the TLR-activating stimuli in bone-marrow-derived macrophages (BMDMs) by increasing the production of inflammatory cytokines (*e.g.*, interferons, IL-6 and TNF-α) and decreasing the production of other regulatory cytokines (*e.g.*, IL-10) (Lebre et al., 2020). This study showed that pristine graphene particles could reprogram the macrophages and induce innate immunity memory that responded to various stimuli through the secretion of inflammatory cytokines (Figure 3). Such plastic products can trigger a long-term impact on innate immunity, produce specific immune memory, and be highly detrimental to human health (Limonta et al., 2019). The effect of MPs on innate immunity memory is an emerging field with potential therapeutic applications that can help to understand the exact mechanism of interaction of plastic products with the human body.

## The immunotoxicity of microplastics

**FIGURE 4:**
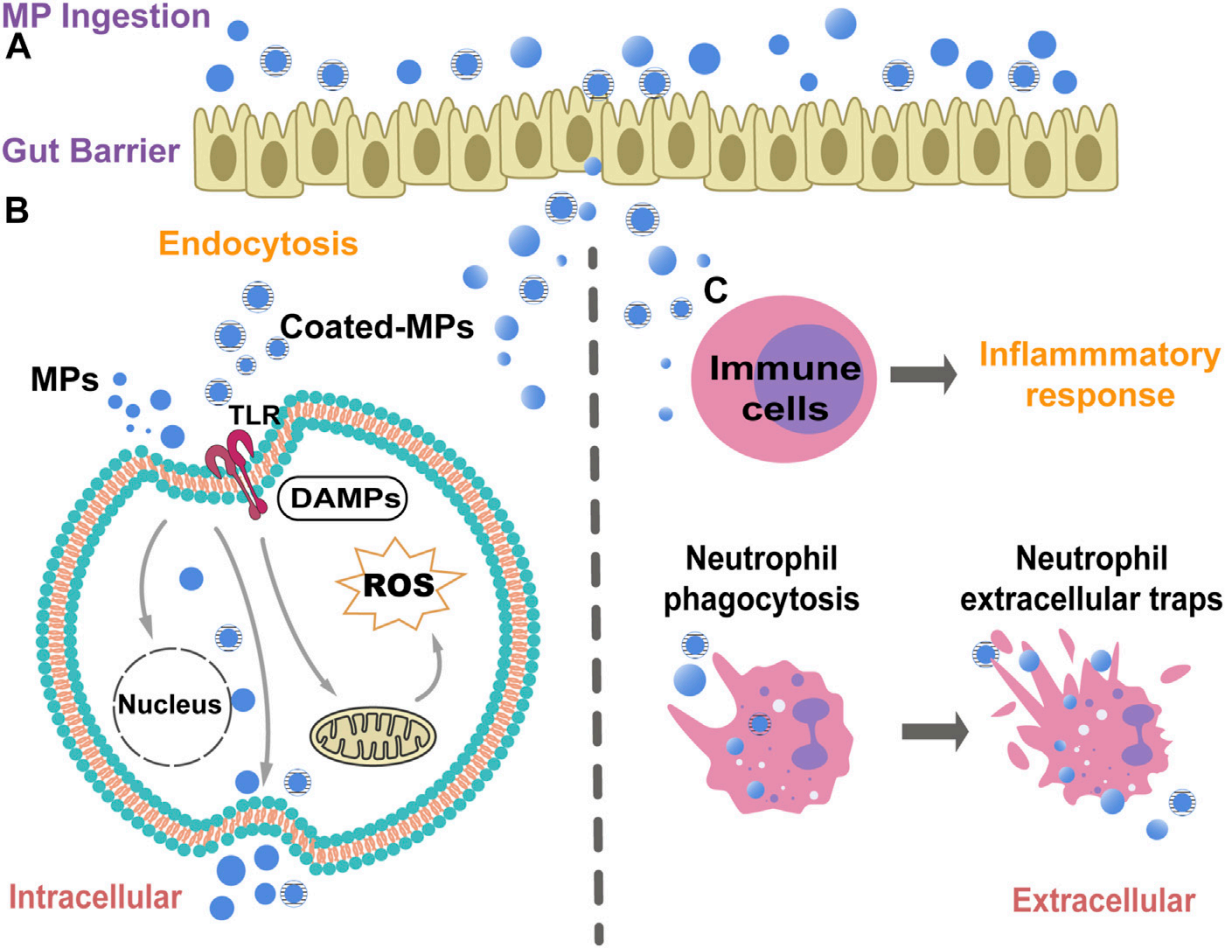
A schematic diagram illustrate the potential biological mechanisms of MPs cytotoxicity and immunotoxicity. **(A)**. MPs can attach to the plasma membrane and infiltrate through the gut barrier after ingestion. **(B)**. MPs can be taken up through endocytosis to affect the potential signaling pathways induction. MPs can induce intracellular ROS and oxidative stress by affecting the mitochondria function, thus finally triggering cellular apoptosis. The MPs could be released out from cells after cell lysis. The coated-MPs may induce the immune responses through the activation of pathogen recognition receptors (PRRs), *e.g.*toll-like receptors (TLRs). **(C)**. The MPs can trigger inflammatory responses after MPs interaction and endocytosis in macrophages and neutrophils. However, massive MPs endocytosis can cause the cell death of neutrophils, which in turn induces the neutrophil extracellular traps (NETs) to capture and restain the MPs in body.

The immunotoxicity of MP uptake by cells mainly arises from oxidative stress via the generation of reactive oxygen species (ROS) and danger-associated molecular patterns (DAMP) (Asharani et al., 2009). ROS produced mainly has two general mechanisms: the impaired mitochondrial respiratory electron transport chain (ETC) and oxidative bursts of NADPH oxidases (NOXs) after the invasion of MPs (Bedard and Krause, 2007). DAMP can induce innate immunity-mediating toll-like receptors (TLRs) and a cascade of inflammatory responses in immune cells (Figure 4) (Gong et al., 2020).

NH_2_-PS MPs negatively affect a wide range of trophic levels by metabolism. The metabonomic analysis of freshwater cyanobacteria metabolism level showed that exposure to NH_2_-PS MPs for 48 h disrupted arginine biosynthesis and glutathione metabolism by down-regulating l-glutamic acid, l-aspartic acid, and l-arginine, and by up-regulating l-glutamine. After NH_2_-PS MP exposure, the increased release of O-phosphoethanolamine induced oxidative stress and membrane damage. This study only investigated the short-term MP exposure, but it provided comprehensive information about the toxic mechanism of freshwater phytoplankton and its potential effects on humans (Feng et al., 2019). With the increasing prevalence of MPs in the environment, there is an urgent *in vitro* and *in vivo* evalutation for their immunotoxic effects on cells and organs, as well as the potential risk to human health. The 20 µm diameters MPs preferentially accumulated in the liver, while the 5 µm diameters PS MPs appeared in the gut more than kidneys after MP exposure to mice (Deng et al., 2017a). The MP-containing mice demonstrated a significant drop in ATP concentration in liver mitochondria and disturbed the lipid metabolism (Mowat, 2003). A disturbed lipid metabolism, altered triglyceride (TG) levels, decreased microbiome, and a drop in body weight in the mice fed with 1,000 μg/L of 0.5 µm and 50 μm PS MPs (Liu et al., 2019). Decreased gut microbiome and mucus production in MPs-feed mice weakened the immunological barrier to microbial infections (Cornick et al., 2015). Rat peritoneal macrophages showed a clear preference for the 1,100 nm particles compared to those with a size of 100 nm, which indicates that phagocytes seem to search for particles that are more comparable to the size of a bacterium (Pratten and Lloyd, 1986). Previous analysis showed that small latex spheres of 300 nm caused platelet aggregation, which could be inhibited by the EDTA chelator (Movat et al., 1965; Pratten and Lloyd, 1986). This implies that MPs exposed to wounds or injured blood vessels may cause more severe damages. Exposure to MPs up-regulated the TCIM (transcriptional and immune response regulator) gene expression, promoting cell proliferation and inhibiting apoptosis to protect body. Moreover, MPs induced in human thyroid and lung cancers by controlling genes and facilitated cancer deterioration (Limonta et al., 2019). In these studies, MP exposure exhibited immunotoxicity, while the immune system responds accordingly to the presence of MPs, which emphasizes the need for more immunotoxicity studies on MPs.

## Conclusion and future perspectives

This review described the potential impact of MPs and other plastic additives on the human immune system and immunotoxicity. Current researches have highlighted the hazardous effects of MPs on macrophages, neutrophils, and other immune cells in humans. However, the immunological mechanisms and pathways after short- and long-term exposure of MPs are still unclear. More research is needed to understand the potentially harmful effects of plastics on human health, and the possible immunological mechanisms of the our body against these plastic particles. Meanwhile, with facing the challenges against the ever-expanding amount of plastics worldwide, the properties of MPs that are hard to be degraded *in vivo* and hard to be specifically recognized by the immune system is another concerning point. Besides, the combined exposure of MPs from enviroment including the combination with bacteria, virus, chemicals and other pollutants, pose a greater risk of MPs on the immunotoxicity.
